# Indirect Potable Reuse: A Sustainable Water Supply Alternative

**DOI:** 10.3390/ijerph6031174

**Published:** 2009-03-17

**Authors:** Clemencia Rodriguez, Paul Van Buynder, Richard Lugg, Palenque Blair, Brian Devine, Angus Cook, Philip Weinstein

**Affiliations:** 1 School of Population Health, Faculty of Medicine, Dentistry and Health Sciences, The University of Western Australia, 35 Stirling Hwy, (M431) Crawley WA 6009 Western Australia, Australia; E-Mails: Brian.Devine@uwa.edu.au (B.D.); Angus.Cook@uwa.edu.au (A.C.); Philip.Weinstein@uwa.edu.au (P.W.); 2 Department of Health, Government of Western Australia, Grace Vaughan House 227 Stubbs Terrace, Shenton Park, WA 6008 Western Australia, Australia; E-Mails: Paul.VanBuynder@health.wa.gov.au (P.B.); Richard.Lugg@health.wa.gov.au (R.L.); 3 Water Corporation, Western Australia, 629 Newcastle Street, Leederville, Perth WA 6007 Western Australia, Australia; E-Mail: Palenque.Blair@watercorporation.com.au

**Keywords:** Chemicals of concern, health impacts, risk assessment, recycled water

## Abstract

The growing scarcity of potable water supplies is among the most important issues facing many cities, in particular those using single sources of water that are climate dependent. Consequently, urban centers are looking to alternative sources of water supply that can supplement variable rainfall and meet the demands of population growth. A diversified portfolio of water sources is required to ensure public health, as well as social, economical and environmental sustainability. One of the options considered is the augmentation of drinking water supplies with advanced treated recycled water. This paper aims to provide a state of the art review of water recycling for drinking purposes with emphasis on membrane treatment processes. An overview of significant indirect potable reuse projects is presented followed by a description of the epidemiological and toxicological studies evaluating any potential human health impacts. Finally, a summary of key operational measures to protect human health and the areas that require further research are discussed.

## 1. Introduction

With climate change, population growth and water scarcity, there is a growing need to manage water resources in a sustainable manner. Worldwide, 1.1 billion people lack access to adequate water supplies [1] and there is an increased pressure on the world’s freshwater sources. Many large rivers, particularly in semiarid regions, have significantly reduced flows and the abstraction of groundwater is unsustainable, resulting in declining water tables in numerous regions [2–4]. Therefore, the use of recycled water has become an increasingly important source of water. Water-recycling projects for non-potable end uses are a common practice with more than 3,300 projects registered worldwide in 2005 [5].

Indirect potable reuse (IPR) is one of the water recycling applications that has developed, largely as a result of advances in treatment technology that enables the production of high quality recycled water at increasingly reasonable costs and reduced energy inputs. In IPR, municipal wastewater is highly treated and discharged directly into groundwater or surface water sources with the intent of augmenting drinking water supplies [6]. In this review paper, recycled water refers to wastewater from sewage treatment plants treated to a level suitable for IPR. Unplanned or incidental use of wastewater for drinking purposes has taken place for a long time. This occurs where wastewater is discharged from a wastewater treatment plant to a river and subsequently used as drinking water source for a downstream community. In contrast, this review focuses on *planned* IPR. The use of environmental buffers such as rivers, dams, lakes or aquifers is considered world’s best practice given that natural systems have a high capacity to further purify water [7]. Retention time of the recycled water in the raw water supply allows any remaining contaminants to be degraded by physical processes (e.g. natural ultraviolet light) or biological processes (e.g. ‘native’ micro-organisms). Storage of the recycled water for a period of time before consumption provides an interval of time in which to either stop delivery of water or to apply corrective actions in the event of a treatment failure. Dilution of recycled water in the environmental buffer also minimizes any potential risk by decreasing the concentration of contaminants that may be present.

Cities with limited water resources are considering IPR as a feasible option for the sustainable management of water because it is a water supply alternative not dependent on rainfall and it is possible to achieve high quality recycled water in compliance with drinking water standards and guidelines. IPR has the potential to make a significant contribution to urban water resources needs but a cautious approach is required to manage the health risk associated with recycled water for drinking. The number and concentration of chemical and biological hazards in wastewater is far higher than the potential hazards that could be found in pristine waters. Contaminants have been detected at low concentrations in highly treated recycled water and any potential health impacts need to be evaluated. Moreover, there are currently no health values for most of these contaminants and usually there are limited toxicological information available. Therefore, an analysis of potential human and environmental risks and the involvement of the community before any implementation proceeds need to be carefully undertaken on a case-by-case basis. This paper presents the “state of the art” context of water treatment, the lessons learned from existing projects and the issues that require further research from the public health perspective. Three supporting tables are provided; Demonstration and full-scale IPR projects (Table 1), Epidemiological studies (Table 2), and Toxicological studies (Table 3).

## 2. Existing Indirect Potable Reuse Projects

IPR is not new and has been successfully implemented in the United States (US), Europe and Singapore. In the US, California is the leading state with the highest number of IPR projects and more than 40 years experience; other states with demonstration or full-scale IPR projects include Arizona, Colorado, Texas, Florida and Virginia. In California, Water Factory 21, in the Orange County Water District (OCWD), is the oldest project, with a production capacity of 19 megalitres per day (ML/day). Water Factory 21 was closed in 2004 and the upgraded groundwater replenishment system (GRS) plant was completed in 2007. The GRS produces 265 ML/day with an ultimate capacity of 492 ML/day [8].

Table 1 provides a summary of 14 well-documented IPR projects around the word. The majority of the projects operate in the US, half of these projects were implemented before the 1980s and four were demonstration plants. The Tampa, San Diego and Potomac demonstration projects aimed to evaluate the feasibility of augmenting drinking water supplies with recycled water, whereas the Denver demonstration project aimed to study the viability of direct potable reuse. The environmental buffers used are mainly aquifers and reservoirs before drinking water treatment. The population served varies from 60,000 inhabitants in the Torreele’s water reuse facility in Belgium to more than 2.3 million in the GRS (OCWD) project.

Other projects in the US have also implemented IPR (not included in Table 1), such as the Gwinnett County Department of Public Utilities, Lawrenceville, Georgia; Inland Empire Utilities Agency, Chino, California; Water Campus, City of Scottsdale, Arizona; El Segundo, California; Tahoe-Truckee Sanitation Agency Water Reclamation Plant, Reno, Nevada; Loe J. Vander Lans Advanced Water Treatment Facility, Long Beach, California; and Northwest Water Resource Centre, Las Vegas, Nevada [9]. All these projects have been supported by their communities and they follow the respective federal or state regulations related to recycled water.

Numerous cities in Europe rely on unplanned IPR for approximately 70% of their potable water source during dry conditions [10]. The IPR project in Wulpen, Belgium, discharges recycled water to an unconfined dune aquifer. Initially the recycled water comprised 90% reverse osmosis (RO) permeate and 10% microfiltration (MF) permeate. However, it was observed that some herbicides were present in the recycled water at levels below drinking water standards due to detection of herbicides in the MF permeate. As a result, since May 2004, only the RO permeate is injected into the aquifer with addition of sodium hydroxide to adjust the pH [11].

In Singapore, a demonstration facility at Bedock Water Reclamation Plant was commissioned in 2000 to evaluate the performance of a dual membrane technology to reliable produce recycled water for IPR and high grade quality water for industry use [12]. Three additional water reclamation plants were commissioned at Kranji (2002) and Seletar (2004) and Ulu Pandan (2007) producing approximately 200 ML/day [13].

In Australia, there are some projects considering the use of IPR through aquifer recharge or dam supplementation, but none as yet implementing potable reuse. IPR has been proposed for Toowoomba (Queensland), Perth (Western Australia), Goulburn (New South Wales) and South East Queensland [14]. In the City of Perth a pilot IPR trial will inject up to 5 ML/day of MF/RO and ultra violet (UV) light disinfected recycled water from the Beenyup Wastewater Treatment Plant (WWTP) into the Leederville aquifer (a major drinking water source for the metropolitan area). If this pilot trial successfully demonstrates no health or environmental impacts, a full-scale project is proposed by 2015 [15]. The City of Goulburn, New South Wales, is also seeking support for a project to supply its dam with recycled water. Goulburn is undertaking lengthy community consultation on all its available water management options, but in 2008 41% of local people surveyed considered IPR undesirable [16].

The Toowoomba project, which aimed to add recycled water to supplement the drinking water supply of the Cooby Dam, did not receive community support in a referendum held in July 2006, with 62% of votes against IPR [17]. Nevertheless, the Queensland Government supported the Western Corridor Recycled Water Project, which included Toowoomba, with a capacity to produce 182 ML/day of recycled water for industrial and potable purposes including supplementation of Wivenhoe Dam [18]. Given the critical water supply situation in Queensland, the community was more sympathetic to the project in late 2007 and early 2008, but due to increased rainfall in the region that increased the dam capacity above 45% they were less supportive in late 2008. As a consequence, despite having built three advanced treatment plants for recycled water, at the end of 2008, the Government changed its recycled water policy from continuous use of IPR to emergency use when dams fall below 40% capacity.

## 3. Studies on Health Effects

Despite variations in treatment technologies, environmental buffers used, proportions of recycled water blended with the raw drinking water sources (from 1% to 100%), and estimated retention times in the receiving waters (from 40 days to several years), none of the projects listed in Table 1 have reported adverse health impacts in the communities served.

In 1998 the US National Research Council (NRC) published the evaluation and recommendations of a multidisciplinary team of experts that explored the viability of augmenting potable water supplies with recycled water. The report concluded that, from the information available, the risk from IPR projects were similar to or less than the risks from conventional sources, but nonetheless considered that IPR should be an option of last resort [7].

### 3.1. Epidemiological Studies

There are few published epidemiological studies on potable reuse and a summary is presented in Table 2. In Windhoek, Namibia, potable reuse was implemented in 1968 and it was initially used sporadically when drought conditions made it necessary. An ecological study conducted in Windhoek examining diarrhoea and type of water supplied concludes that differences in diarrhoeal disease prevalence was associated with socio-economic factors, but not the nature of the water supply [7]. So far, no studies have been conducted in the Windhoek project examining long-term potential health impacts of micropollutants in drinking water.

In the Montebello Forebay project, three epidemiological studies were published, two of them using an ecological design. The latest ecological study was published in 1996 (Table 2). In this study, a significantly higher incidence rate of liver cancer in the area with the highest percentage of recycled water was observed. However, no significant trend was observed when comparing liver cancer incidence over different exposure categories, and the authors concluded that the positive association occurred by chance. The study does not provide evidence that recycled water has an adverse effect on cancer incidence, mortality or infectious disease outcomes. However, the ecological studies performed thus far have been limited by their design and the corresponding difficulties that arise in the accurate assessment of the exposure [19]. A cohort study examining the association between the use of recycled water and adverse birth outcomes, including 19 categories of birth defects, was conducted from 1982 to 1993. This study did not find any significant association between the use of recycled water and adverse birth outcomes, and rates were also similar in groups receiving high and low proportions of recycled water [20].

No prospective studies have been conducted examining the potential adverse health effects of long-term exposure to low concentration of chemical contaminants from potable reuse. However, assessment of exposure is especially challenging in studies with long latency periods, such as cancer. In the late 1990s the OCWD and an independent scientific advisory panel suggested conducting a case-control study on the use of Santa Ana River water. However, the study was found to be non-feasible due to limitations in assessing historical exposures. The panel did not recommend any additional epidemiological studies because any incremental risk due to recycled water is likely to be extremely small and difficult to differentiate from normal background risk [21]. The panel instead recommended a focus on monitoring to verify the effectiveness of the treatment processes.

Given that epidemiological studies of long latency (such as cancer outcomes) are associated with many competitive risk factors and are complicated by limitations in the assessment of the exposure, epidemiological studies with health endpoints of short latency (such as gastrointestinal diseases or adverse pregnancy outcomes) may be more appropriate as a means of elucidating possible disease pathways. A critical aspect for projects considering the implementation of epidemiological studies is the need to carefully assess the exposure to recycled water in the study population during the period of interest. Hydrogeological modeling, geographic information systems and exposure data at the individual level may be required to link health outcomes with levels of exposure to recycled water.

### 3.2. Toxicological Studies

Toxicological testing is the primary component of chemical risk assessments of IPR projects. Estimations of human health risks from exposure to specific chemicals are generally based on extrapolations of toxicological analyses on animals. Given that toxicological information exists only for a small percentage of chemicals and that toxicological data for individual compounds are not adequate for predicting risks posed by chemical mixtures, it is usually the concentrates of recycled water which have been used to assess potential health risks [13]. Overall, toxicological studies have varied in approach and study aims, but no significant health risks have been identified from these studies (Table 3).

In the US, only the Denver and Tampa studies assessed a wide range of toxicological endpoints. These studies included sub-chronic and chronic toxicity testing, as well as specific health effects (such as reproductive, developmental and carcinogenic outcomes). In these two demonstration projects and in Singapore, toxicological analyses have been performed by comparing the health effects on animals (usually rats and mice) fed over several generations with recycled water concentrates, compared with control groups. The Denver report concludes “no adverse health effects were detected from lifetime exposure to different concentrate samples during a two-generation reproductive sample” [22]. In Singapore, the health effects testing programme also concluded that exposure to, or consumption of, recycled water does not have carcinogenic or estrogenic effects on fish or mice [23]. Finally, the Tampa study did not report any increased adverse health effects on animals fed with recycled water.

Mutagenic studies using the Ames test, which is used to determine whether a chemical is able to cause cell mutations to the bacteria *Salmonella typhimurium*, were performed in the San Diego, Tampa, Potomac Estuary, OCWD and Montebello Forebay projects. In general, less mutagenic activity was observed in recycled waters compared to other water sources. In the Montebello Forebay project, mutagenic activity was detected in 43 of the 56 samples from both recycled and control waters tested. The observed level of mutagenic activity was maximal for storm runoff, but lower (in declining order) for dry weather runoff, recycled water, ground water and imported water [24]. The Ames test is a commonly used screening tool and is easy to perform, but may produce a relatively high proportion of false positives and false negatives. Most of the mutagenic activity that was found appeared to be linked to the chlorination process. However, identification of specific mutagens was not possible due very low concentrations of contaminants but the National Research Council recommended further studies to characterize the chemicals involved in the mutagenic activity of the recycled water given the consistency of findings among the evaluated studies [7].

Bioassays conducted for estrogen, androgen, and thyroid activity have shown a progressive endocrine activity reduction during the treatment train and a very low endocrine activity in the product water [25]. Lee *et al.* reported low estrogenic activities (measured as estradiol equivalent concentrations, or EEQ) of 0.23 and 0.05 ng-EEQ/L after MF and RO respectively. The estrogenic activities were at markedly reduced values compared with the value of 1.2 ng-EEQ/L in the plant influent. The bioassay EEQ measurement and the EEQ calculated from chemical analysis of known estrogenic chemicals were similar for samples taken both after MF and after RO. However, the EEQ in the influent was twice as high when calculated by chemical analysis compared with the bioassay, due in part to antagonistic effects between chemicals. Consequently, the removals of endocrine disrupting compounds in terms of the EEQ value from the biological and chemical determinations were 80 and 96% for MF and RO respectively [26].

## 4. Measures for Public Health Protection

A variety of factors must be carefully assessed to ensure public health protection. Some of the fundamental practices and lessons learned from the implementation of IPR projects are presented in this section. These factors include the treatment processes required to achieve high water quality; the quality of the existing water supply and any changes in this source after recycled water is blended; system reliability; the regulatory framework and risk management practices.

### 4.1. Recycled Water Quality and Monitoring

Analytical monitoring programs of existing IPR projects listed in Table 1 have demonstrated the effectiveness of advanced treatment in meeting all primary and secondary drinking water standards. For example, in the NeWater project in Singapore, more than 190 drinking water parameters are monitored, and the project consistently meets the requirements stipulated in the USEPA and WHO drinking water guidelines [23]. Furthermore, all projects described in Table 1 have reported that the treatment can reliably produce water of equal or better quality than that of the existing untreated or treated drinking water supplies [21–23,27–29]. It is accepted that advanced treatment can produce recycled water in compliance with drinking water standards and guidelines. Although this compliance is fundamental to the protection of public health, it does not necessarily guarantee the safety of the recycled water. Wastewater often comprises a complex mixture of domestic, industrial and agricultural contaminants. Therefore, monitoring for contaminants either known or suspected to be present in wastewaters at concentrations of concern needs to be implemented to demonstrate that the concentrations of these contaminants, if present after the treatment, do not pose any additional health risk.

Characterization of biological and chemical agents in the product water has been carried out in all projects described in Table 1. Despite variations in treatment technologies and technological changes over time, all IPR projects have demonstrated high removal efficiency for contaminants tested. Removal of unregulated chemical contaminants was tested in the San Diego and Denver demonstration plants [22]. In Denver, an organic challenge study tested the treatment efficiency in removing chemicals. Fifteen organic compounds were dosed at 100 times the normal levels found in the treatment plant influent, and the results demonstrated that the multiple-barrier process could remove those contaminants to non-detectable levels [22]. In San Diego, the monitoring program demonstrated the effectiveness of RO in removing metals, other inorganic compounds, and 29 pharmaceuticals and personal care products, including caffeine and ibuprofen, typically found in wastewater from secondary treatment plants [30]. Testing for non-regulated contaminants such as endocrine disrupters, pharmaceuticals and personal care products is currently underway in many projects as part of regulatory requirements or research interest. For example, in the GRS (OCWD) project, concentrations of estrone, 17-α-ethynyl estradiol and 17-β-estradiol were all below the detection limit of 10 ng/L, and caffeine concentration was below 0.1 μg/L in the recycled water [8].

Various guidelines suggest that the minimum log reductions required for IPR are: 8 log for *Cryptosporidium,* 9.5−10 log for enteric viruses and 8 log for *Campylobacter* [61]. MF is able to remove protozoan oocysts and cysts, algae and some bacteria and viruses [31]. Viruses are the biological contaminants of major concern in IPR, due to the large numbers present in wastewater and their small size (range from 0.01 to 0.1 microns). Because pathogenic viruses have the potential to cause disease outbreaks from a single spike of exposure, they are a high public health priority. MS2 bacteriophage has been used to validate membrane performance. MF alone produced a 1.9 log removal of MS2 bacteriophage [32] and ultrafiltration and RO can provide 2 to 6 log removal [33,34] MS2 has been detected in RO permeate as a result of faults or damage in membrane structure [35]. In addition, variable log removal has been reported with variable influent concentrations of MS2 [35] and the MS2 sensitivity to (UV) light was not constant [32]. These issues are complicated by difficulties in isolating and measuring viruses and the cost of the analysis. The removal of virus by MF/RO is dependent upon the particular membrane being employed and therefore the estimation of the removal or inactivation credit for viruses ideally should be done on a “membrane by membrane” basis. Therefore, projects considering IPR need to: identify membrane manufacturer studies to remove pathogens with special relevance to virus, validate the treatment process using accredited methods and protocols; perform suitable challenge tests for viruses to ensure the treatment efficiently removes these contaminants and verify the integrity of the membrane systems through routine testing. Direct methods of membrane testing, such as the pressure hold test and the diffusive air flow test, are very sensitive to identify impaired membrane integrity but they cannot be applied while the plant is in operation. Indirect methods such as particle counting, turbidity and conductivity are less sensitive but are continuous and online, and can be used as surrogates to monitor membrane integrity. Therefore a combination of both direct and indirect methods is recommended for a comprehensive monitoring program [34].

Chemicals that have been detected in secondary effluents include household and industrial chemicals such as detergents, flame retardants, plasticizers, personal care products and pharmaceuticals. Some of these compounds are known or suspected carcinogens, others are estrogenic and have the potential to adversely affect the endocrine system. Advanced treatment technologies such as MF/RO followed by advanced oxidation processes and/or UV are able to remove most of these contaminants to levels below limits of detection (ng/L) [36–38]. It is important to note that organic contaminants have also been detected in many other drinking water sources at low concentrations (< 0.1 μg/L). The US GS Water Quality Assessment Program has determined that streams and rivers used for public drinking water have low levels of about 130 chemical contaminants, most of them without drinking water standards. Nearly two-third of these contaminants were also found in drinking water. These results indicate that conventional drinking water treatment was unable to remove the trace contaminants, and that unplanned potable reuse (as currently happens in many places in the world) has the potential to result in large concentrations of micropollutants in drinking water supplies. The most commonly detected chemicals were herbicides, disinfection by-products, and fragrances. A median of 4 to 6 compounds were detected per site indicating that the targeted chemicals generally occur in mixtures and that they originate from a variety of household and industrial sources [39,40].

Many IPR recycled water projects implement monitoring programs to evaluate the treatment efficiency in rejecting organic contaminants, including endocrine disrupters, pharmaceuticals and personal care products and other unregulated compounds. Antibiotics are of special interest because of growing concerns over antimicrobial resistance in human medicine. Disinfection by-products may be generated during the treatment process and some of them can be stable, polar and toxic, such as N-nitrosamines and trihalomethanes. Their formation should be avoided or their removal accomplished as far as possible in any potable reuse project. Endocrine disrupters (particularly those with an estrogenic effect) produce adverse effects in fish and other species at low concentrations. Within the framework of the precautionary principle, the reliability of advanced treatment in removing such compounds to the maximum extent achievable needs to be demonstrated for the protection of human health.

Drewes *et al.* recommended the use of chemical indicators and surrogates to monitor treatment performance. They selected a list of wastewater-derived contaminants to determine the treatment removal efficiency of individual unit processes commonly used in IPR (i.e., soil aquifer treatment, ozone, advanced oxidation, chlorination, carbon adsorption, and RO). The authors validated the removal efficiency of the selected chemicals for each unit process through laboratory, pilot, and full-scale experiments. Different groups of chemicals, sharing similar physicochemical characteristics, were detected at low concentrations (ng/L) for each one of the unit processes. The report concludes that, by selecting multiple chemical indicators with different physicochemical properties, it is possible to account for compounds currently not identified and new compounds synthesized and entering the environment in the future, provided they fall within the range of properties covered. The underlying concept is that absence or removal of an indicator compound during a treatment process would also assure the absence or removal of other compounds with similar properties. For example, the authors recommended the use of sulfamethoxazole, *N*-nitrosodimethylamine (NDMA), tris(2-chloroethyl)-phosphate (TCEP) and chloroform as chemical indicators during the initial phase of the IPR project and the use of conductivity, total organic carbon (TOC), and boron as surrogate parameters for the MF/RO system [38].

### 4.2. Membrane Treatment and the Multiple Barrier Approach in Treatment

Ultrafiltration or MF as pre-treatment for RO followed by UV treatment or advanced oxidation are the commonly used treatment steps in IPR. Secondary effluent from conventional wastewater treatment plants is treated by MF, which is a low-pressure membrane with a pore size of 0.01 μm. MF can remove most of the fine suspended solids (more than 99% rejection), colloidal solids, bacteria and protozoa. [29,41–43]. After MF the water passes through the RO, a high-pressure process that forces water through the porosity matrix of a specialized membrane. RO can reject high molecular weight organic matter (characterized as humic and fulvic acids) [44] and total organic carbon rejection is normally higher than 96% [28]. Removal of biochemical oxygen demand and chemical oxygen demand has been reported as high as 98% and 96% respectively [28]. RO separates out minerals and other contaminants, including heavy metals, viruses, and pesticides [43,45].

In the studies conducted so far, high percentages of organic contaminant removal are commonly reported. RO can remove up to 95 to 99% of hormones [36,46], and more than 95% of all tested analytes, including 16 pharmaceuticals and three personal care products [47]. In general, membranes are able to reject most of the endocrine disrupters, pharmaceuticals and personal care products, with the exception of lower molecular weight compounds [48,49]. However, incomplete rejection of certain disinfection by-products, and some micropollutants of low molecular weight has been reported during full and pilot scale high-pressure membrane applications [50]. Organic chemicals of high molecular weight are effectively rejected by the MF/RO treatment, but those of low molecular weight (less than 500 Dalton) are less effectively rejected and have been detected in the RO permeate at low concentrations [51]. However, the low molecular weight compounds detected in product water are present in trace concentrations well below health significance.

As in drinking water treatment, the multiple barrier approach is also used in IPR. The approach includes source control, use of multiple water treatment processes, use of environmental buffers and conventional drinking water treatment. The basis of this approach is to ensure that there are several independent steps in place to remove contaminants given that no single barrier is able to remove all contaminants from wastewater. The multiple barriers also minimize the risk by producing less variation in the final water quality and by providing some protection in the event of poor performance of one barrier, provided some degree of adjustment can be achieved in other treatment barriers to compensate for temporary failures (e.g. disinfectant doses can be increased if membrane filtration underperforms).

Source wastewater assessment and protection is the first barrier and it is critical to prevent contaminants from entering the wastewater. Source control requirements should be part of the formal approval process to utilize recycled water for IPR as such requirements identify and minimize the introduction of contaminants into the wastewater, minimising the need for them to be removed through treatment. In Australia, the National Waste water Source Management Draft Guideline provides a framework for good management of the quality and quantity of all wastewater source inputs to a wastewater collection, transfer, treatment and disposal/reuse systems. The framework has been ordered into five key wastewater input management objectives which cover the quality of all possible source inputs with the potential to impact on sewage quality. These objectives address protection of safety in sewers, infrastructure assets, treatment plants, regulatory compliance and recycling [52]. Therefore, government agencies responsible for industrial wastewater control programs, as well as relevant stakeholders, need to periodically review discharge permits, inspections programs, wastewater monitoring plans, and enforceable discharge standards. Additional barriers beyond the advanced treatment process include retention times in aquifers or surface waters as they act as an extra barrier, as a buffer, to provide time to initiate corrective actions if required followed by drinking water treatment before distribution to the community.

For the protection of human health, each treatment process must be evaluated to establish its performance against the different categories of contaminants. A timely and effective monitoring program is fundamental to detect the unexpected appearance of contaminants in the recycled water. For example, additional treatment barriers after RO were implemented in the GRS (OCWD) project after the detection of NDMA and 1,4 dioxane, both of which are potentially carcinogenic [29]. An advanced oxidation process using hydrogen peroxide and UV radiation where added to break down these contaminants and other potential undetected organic compounds [29].

### 4.3. Regulatory Framework

Different regions using IPR have developed various approaches to ensure health and environmental protection. In the US, there are no federal regulations governing IPR and criteria are developed at the state level. Therefore, states operating IPR projects, such as California, Washington, Arizona and Florida, have each developed various guidelines. Criteria among states are generally similar and tend to be conservative with an emphasis on maintaining protection of public health [53]. In California, recycled water regulations for groundwater recharge of potable aquifers requires secondary treatment, filtration, disinfection, and advanced wastewater treatment. Water quality goals, at that time, included: pH 6.5–8.5; turbidity less than 2 nephelometric turbidity units; no detectable faecal coliforms; less than 1 mg/L chlorine residual, TOC less than 1.0 mg/L; and compliance with all drinking water standards [54]. In Florida, IPR projects have to meet drinking water standards: TOC less than 3.0 mg/L, total organic halides less than 0.2 mg/L, and total nitrogen less than 10.0 mg/L [53,55].

Recycled water guidelines include both monitoring and performance requirements [56]. The Department of Health Services, now the California Department of Public Health (CDPH) released the first draft criteria for IPR via groundwater recharge in 1986. These guidelines revised in 2008 are considered the most developed so far in the US, and include monitoring requirements related to nitrogen compounds, unregulated emerging chemical contaminants (such as endocrine disrupters and pharmaceuticals), and TOC limits [57]. The latest groundwater recharge reuse draft released by the CDPH in August 2008, includes annual monitoring for endocrine disrupting chemicals and pharmaceuticals. Some contaminants are listed in the Endnote No 5 of the Draft Guidelines, although no specific indicator chemicals are recommended [58]. TOC requirement depends upon the degree of recycled water recharged and should not exceed 0.5 mg/L divided by the proposed maximum recycled water contribution [58]. CDPH is continually updating the guidelines as more information becomes available. No doubt regulation will continue to evolve to address new issues or concerns as they arise. Each project needs to select the contaminants to be included in its ongoing monitoring program based on wastewater characteristics, treatment processes and risk assessments. Ongoing monitoring is recommended to identify reliable indicator or surrogate chemicals. In 2007 the CDPH published a Treatment Technology Report for Recycled Water identifying the recognized technologies that were acceptable for compliance with treatment requirements [54]. RO is required for all IPR injection projects and the minimum retention time in the aquifers is set at 12 months for direct injection and 6 months for infiltration of recycled water through soil.

Recycled water guidelines are now incorporating several approaches using a risk management framework to ensure minimum levels of risk and maximum quality of the final product water. Best Available Technology [59], Life Cycle Analysis and Hazard Analysis and Critical Control Points (HACCP) [60,61] are some of the more commonly used approaches. The HACCP concept was originally developed for risk management decisions involving health and safety in food and later used in the pharmaceutical industry [62–64] and has been introduced for drinking water [60] and recycled water [61,65,66]. The HACCP approach was used in the Australian Drinking Water Guidelines [67] and in the National Guidelines for Water Recycling Phase 1 [68]. These latter guidelines include a risk management framework and specific guidance on managing the health risks associated with the use of recycled water for all applications other than potable use. The guidelines are intended to provide a unified approach across Australia. The Phase 2 Guidelines for Water Recycling: Augmentation of Drinking Water Supplies was released in May 2008 and they also follow a risk management approach to ensure health protection [6].

The HACCP approach includes hazard identification and risk assessment, identification of appropriate preventive measures, and operational monitoring of the preventive measures. The aim of operational monitoring is to measure ongoing performance of preventive measures and to ensure that, where required, corrective action is implemented prior to the water being released. In some cases, monitoring can be continuous, whereas in other contexts, discrete sampling at lower frequencies is employed. Because the efficiency of the treatment is variable and depends primarily on the quality of the influent water the pressure of the water through the membranes, and the porosity of the membranes [69], a well-designed treatment process is essential to ensure adequate system reliability and satisfactory operation over its lifetime.

Compliance testing alone is not enough to protect public health [70]. Firstly, it is not practical to test for a large set of contaminants, as data gathering is costly in both time and resources. Furthermore, analysis of the water quality is time-consuming and non-compliance with guideline values is always detected *after* contaminated water has already been supplied; that is, it constitutes a “retrospective” assessment. Many contaminants present at low concentrations are not directly or easily measurable. Therefore, a coherent and structured evaluation of the hazards, and the management of the critical control points plays a central role in the safe operation of recycled water projects. Consequently efforts to protect public health should focus on failure detection systems that measure the performance of key process units rather than just monitoring the final effluent or the end-use point. For example, the parameters to identify failures in the performance of the MF and RO processes are generally indicated by turbidity and conductivity respectively, that can both be monitored continuously using appropriate plumbed-in instrumentation.

In summary, in order to conform to the HACCP management approach, very stringent water quality and monitoring requirements are imposed for IPR. Typical requirements include advanced treatment of the secondary effluent using MF/RO and in some cases also UV and/or advanced oxidation processes to remove chemical and biological hazards, conformance with drinking water guidelines in the product water, extensive monitoring for known or suspected contaminants, and minimum residence time in the receiving aquifer or surface water body. Other requirements include monitoring and site-specific controls on the operation, maintenance and management of the plants.

## 5. Knowledge Gaps, Aspects to be Implemented and Future Research

### 5.1. Recycled Water Quality, Monitoring and Risk Assessment

Analytical methods have been developed for a wide variety of compounds and isotopically labelled standards have become commercially available in recent years. However, large-scale method comparison and validation exercises to improve the accuracy and precision of quantitative measurements have not yet been conducted. It is currently difficult to interpret and compare treatment efficiency in the removal of emerging contaminants. More research is needed not only to identify new potential contaminants of concern in recycled water, but also to develop validated methods and implement harmonized analytical methods. Validated methods for emerging and other unregulated contaminants will: (i) facilitate the risk assessment and regulatory process by providing better quality data; (ii) provide comparative information about contaminant fate and removal during the treatment barriers; and (iii) assist the analysis of different treatment options for removing contaminants. In 2005, the 6^th^ European Union framework funded the Norman Project, which aims to create a network of reference laboratories and related organizations for chemical monitoring and biomonitoring of emerging environmental pollutants [71–73]. In future years, it is expected that progress will be made in the validation and standardization of chemical analysis and biomonitoring techniques for recycled water relating to emerging pollutants.

A greater research focus to manage health risks from trace organic compounds in recycled water is needed, with a particular emphasis on investigating the toxicological relevance of endocrine disrupters and pharmaceuticals in recycled water. The impact of endocrine disrupters in fish and other species exposed to wastewater have been documented [74–76], but the implications of these findings for human health remain inconclusive. There is also a need to develop approaches on recycled water traceability that would permit attribution of the proportion of recycled water used in the context of risk assessment and management studies. Given that it is not practical to test for a large set of chemicals of concern, it is also essential to identify appropriate tracer or indicator compounds to follow their occurrence and removal in the validation, verification and ongoing monitoring programs.

Validated monitoring approaches are required to ensure adequate health protection for a number of reasons: (i) several unregulated chemicals of concern are not routinely included in monitoring programs; (ii) many emerging chemicals of demonstrated or suspected health concern as yet have no standard analytical methods; (iii) some current analytical methods have detection limits above the toxic threshold; (iv) the possibility of other unknown toxic chemicals in the recycled water; and (iv) combinations of toxic chemicals may exert mixture effects that remain poorly characterized.

Various monitoring approaches are available or in development, but are not in use with IPR projects, include:

1. On-line biomonitoring systems using fish have been developed in recent years to evaluate potential health impacts without using concentrates of recycled water [77]. Behavioral and/or physiological stress responses of organisms exposed in situ are evaluated, to provide additional assurance that untested or as yet undetected chemicals of concern would not remain undetected.
2. Biomarkers for endocrine, developmental, and potential reproductive effects in aquatic organism exposed to recycled water are also under development and seem to be a promising area [78].
3. On-line sensor technologies for triggering contaminant warning systems have proven feasible in the laboratory. For example, the USEPA studied 20 on-line commercial sensors for their ability to identify 25 injected contaminants into the distribution system by testing of 17 water quality parameters. They found that free chlorine and total organic carbon detected the widest array of contaminants and produced the largest, and most easily detectable, water quality changes [79]. However, more research is needed linking changes in physico-chemical water quality indicators to the presence of contaminants relevant in the IPR context, and on the sensitivity and long term reliability of online sensors (such as particle counters).
4. Quantitative structure activity relationship (QSAR) methods are being used not only to predict the potential toxicity of compounds based on their physical and chemical properties [80,81] but also to predict rejection of micropollutants such as pharmaceutically active compounds by different types of membranes during IPR treatment. This is a promising area that requires further research.

Potential human health effects of previously untested contaminants may necessitate additional regulations. It is fundamental to establish whether these emerging contaminants of concern may pose an additional risk to human health at the concentrations reported in recycled water. A systematic approach is required to evaluate the measured concentrations of contaminants in recycled water against benchmark values [6,82]. This approach may help regulators to identify contaminants that require further health risk assessment or toxicological studies, as well as facilitating communication of study findings in an effective manner to the community.

### 5.2. Regulatory Framework

Although many water authorities are aware of research into the various treatments and contaminant rejection fractions personnel must also be provided with ongoing training in the emerging technologies in IPR. More research and reports are expected in the future regarding the operation of IPR projects and the implementation of management systems, such as the HACCP approach. Moreover, monitoring of parameters, both online, and in the laboratory will identify performance compliance and when threshold values are exceeded enable emerging problems to be detected and corrective actions taken.

Separating drinking water from sewage was a major achievement in the conquest of infectious diseases, and remains a challenge to be overcome in much of the developing world. Now with the intentional and planned augmentation of drinking water supplies with recycled water, it is fundamental to ensure that the community remains protected. Therefore, there is a need to integrate both recycled water and drinking water at the regulatory level. It is not enough to rely on drinking water standards and guidelines to ensure the safety of the recycled water. This may result in a modification to the approach to dealing with emerging contaminants in regulation of traditional drinking water sources. It is also possible that additional chemicals may need to be monitored at drinking water treatment plants once recycled water is introduced to the source water catchment. It is expected that with the continuous development of treatment technologies, analytical methods, monitoring techniques, toxicological studies and risk assessment approaches, the use and regulation of recycled water for IPR will continue to evolve.

### 5.3. Epidemiological Surveillance

Regulators approving IPR projects need to implement a well-coordinated public health surveillance system to document possible warning signs of any adverse health events associated with the ingestion of recycled water. Existing surveillance systems, such as those for notifiable communicable diseases, should be used and/or enhanced to meet these needs. Surveillance systems must be jointly planned and operated by health departments, water utilities and other relevant agencies. Key individuals in each agency need to be appointed to coordinate planning and rehearse emergency procedures. The surveillance plan, its purpose, the monitoring results, and the system process performance should be available to the community and interested stakeholders. Surveillance systems may indicate whether an epidemiological study is required. However, epidemiological surveillance is considered relatively slow and is reactive as it is based on disease outcomes.

In addition to the health surveillance program, the research capacity in regions considering IPR needs to be enhanced to implement a monitoring program that provides an early warning system of potential health risks from newly detected or emerging contaminants. In order for monitoring systems to be effective, a multi-institutional commitment is required for the documentation and monitoring of all significant chemical wastewater inputs from household, commercial, agricultural and industrial sources. Pre-established risk mitigation measures also need to be in place.

### 5.4. Public Perception

Although communities have accepted recycled water for non-drinking purposes such as irrigation of parks, they are less likely to accept the use of recycled water as a drinking water source. The perceived decrease in temporal and geographical distance between wastewater and recycled water in IPR raises reservations amongst the community about the safety and quality of the recycled water. Emotions, or the ‘yuck’ factor, play a major part in people’s lack of acceptance. Nevertheless, increased community support has occurred in the last decade and important progress has occurred in identifying factors of success or failure in the implementation of IPR projects [83–85]. Five aspects were identified by the Water Environment Foundation for building and maintaining community support in recycling projects: “(1) managing information for all stakeholders; (2) maintaining individual motivation and demonstrating organizational commitment; (3) promoting communication and public dialogue; (4) ensuring a fair and sound decision-making process and outcome; and (5) building and maintaining trust” [83]. Promoting communication and public dialogue, and building and maintaining trust have also been identified as key aspects in other studies [86–88].

Effective communication between the community, key stakeholders and the project proponent is crucial to achieve community support. All recycled water projects need to be accompanied by community education to demonstrate that the current technology is adequate to protect human health. A timely and active communication program to discuss the treatment processes, the risks, the measures in place to control risks and the safety of the water, may help to increase trust in the project. The experience in the US has indicated that community understanding and acceptance may take several years, but that a broad community communication approach is fundamental for the successful implementation of IPR projects. There are many examples where local communities have rejected IPR proposals because they were poorly informed or insufficiently confident in the process. Some examples include the Dublin San Ramon Services District in California [9,85] and the Water Futures Toowoomba in Queensland [17], where there was a lack of coordination between the authorities involved in planning, health, water supply and environment, and/or inadequate community consultation on the issue.

Community attitudes to water recycling are dependent on numerous factors, including the degree of water scarcity, the supply costs, the quality of the consultative processes, the perceived management of health risks, and the accountability of, and trust in, the regulator, the government and the water utility. Therefore targeted social research is needed in communities where IPR is proposed to understand the influence of psychological factors related to: perception of risk, motivations, attitudes, beliefs and behavior on the use of recycled water to supplement existing water supplies and IPR project acceptance.

## 6. Concluding Remarks

IPR has been in practice for over 30 years. The projects presented have used advanced treatment technologies and applied the treated recycled water into an environmental buffer where naturalisation and dilution occur. Although few epidemiological studies have been conducted, there is no conclusive evidence that communities using drinking water supplemented with recycled water are at any increased risk of disease compared with those who do not drink recycled water.

IPR is a viable option for supplying reliable potable water to those urban regions with increased water demand and/or decreasing alternative supplies. The use of IPR needs to be evaluated in conjunction with other potential water supply alternatives, and the potential health impacts need to be carefully considered before implementation. This process requires an understanding of how water quality and health standards can be maintained through rigorous controls and monitoring techniques, based on sound science and proven treatment technologies. IPR projects need to demonstrate effectiveness of available barriers, guarantee safety by on-line monitoring systems, process control, testing, and source control programs.

No water treatment is ever without risk, including conventional drinking water treatment and traditional drinking water sources. Similarly, IPR as a new water source will never be a totally risk-free practice. However, using best available technologies, risk assessment and risk management practices, water agencies, health regulators and other stakeholders can evaluate and mitigate the potential public health risks from the biological or chemical contaminants found or likely to be found in the recycled water. Mitigation of hazards to their acceptable risk is critical to balance public health protection and resources. Risk reduction below the acceptable risk will not result in significant reduction in risk and at the same time additional expenditure of resources will not result in significant advances towards increased safety.

It is essential to maintain ongoing research in hazard mitigation and control in IPR projects, coupled with appropriate toxicological and epidemiological studies. The reviewed literature supports the practice of IPR as a reliable and safe addition to existing drinking water supplies, and it is anticipated that IPR will represent an essential element of sustainable urban water resources management in many more regions of the world in the future.

## Appendix

**Table 1.** Demonstration and full scale potable reuse projects.

Project	Place	Year	Treatment	Buffer	Population	% Blended	Comments	Source
Orange County Water District (OCWD). Water Factory 21	California (USA)	1975–2004	Lime clarification, recarbonation, multimedia filtration, granular activated carbon, filtration and chlorination. RO added in 1977. Advanced oxidation with hydrogen peroxide and UV added in 2001	Aquifer	Less than 2 million	3.2% total OC water 4.8% OC groundwater	Full-scale project Water Factory 21 was built in 1975 and decommissioned in 2004. First project that used recycled water to maintain a seawater intrusion barrier. More than half the injected water flows inland and augments potable water supplies. The injected water reaches the nearest drinking water bore after 2 to 3 years. Addition of RO in 1977 enabled injection of up to 50% of recycled water.	[28]
OCWD Groundwater replenishment system (GRS) (Upgrade of the Water Factory 21 plant)	California (USA)	Pilot plant from 2004 to 2007 Full scale plant since 2007	MF/RO and advanced oxidation (UV and hydrogen peroxide)	Aquifer	2.3 million (300,000 to 700,000 additional residents projected by 2020).	15–18%	Demonstration project conducted before construction of the GRS plant produced 5 mgd. Full scale plant produce 70 mgd per year (10% of Orange County's drinking water supply) Initially 75% of the recycled water injected, later 100% injection The groundwater basin supplies more than half of the population water needs.	[8,89]
Denver Potable Water Demonstration Project	Colorado (USA)	1985–1992	Treatments tested included: high-pH lime clarification, sedimentation, recarbonation, filtration, selective ion exchange for ammonia removal, UV irradiation, activated carbon adsorption, RO, air stripping, ozonation, chlorine dioxide disinfection, ultrafiltration and chloramination.	NA	NA	NA	The project investigated different options for alternative water supplies and concluded that potable reuse is a viable option. Pilot plant used unchlorinated secondary effluent from the Denver Wastewater Treatment Plant.	[22]
West Basin Municipal Water District	California (USA)	Since 1995	MF/ RO UV and advanced oxidation processes	Aquifer	950,000	10–15%	Full scale project which produces three types of tertiary treated recycled water for industrial and irrigation uses, and three types of RO water. Softened RO water for groundwater recharge, Pure RO water for low pressure boiler feed, and ultra-pure RO (which has a second pass RO) water for high pressure Ground water recharge represents 22% of the total production. About 75% of the recycled water injected	[90]
Upper Occoquan Sewage Authority (UOSA)	Virginia (USA)	Since 1978	Lime clarification Two-stage recarbonation Flow equalization Sand filtration Granular activated carbon Ion exchange Post carbon filtration Chlorination	Reservoir	1.2 million	10–45 %	Full-scale project. Supplies about 50% of the population’s water supply. During drought periods recycled water provides up to 90% of the reservoir inflow. Recycled water is monitored by an independent water monitoring agency and is considered the most reliable source of water in the Occoquan system.	[91]
Montebello Forebay Groundwater Recharge Project	California (USA)	Since 1962	Secondary treatment, chloramination and injection. Inert media filtration was added in 1977 as an additional measure for public health protection to enhance virus inactivation.	Aquifer	1.28 million	18.7% up to 35%	Full-scale project comprising three plants located in the central basin of Los Angeles County. Whittier Narrows WRP (built 1962) serves approx 150,000 people. The San Jose Creek WRP (built in early 1970s) serves 1 million and Pomona WRP (built in early 1970s) serves 130,000 people. The recharged water is composed of recycled, storm and imported waters. Injection of up to 50% recycled water is acceptable in any given year providing that the running three year total does not exceed 35% of the recycled water.	[19,20, 28]
Tampa Water Resource Recovery Project	Florida (USA)	1987–1989	Pre-aeration, lime clarification, recarbonation, gravity filtration, and ozone disinfection. Granular activated carbon, RO, and ultrafiltration, were also evaluated after filtration and before disinfection.	Reservoir	NA	NA	Demonstration project to evaluate the treatment efficacy of four advanced water treatment processes. Augmenting the reservoir with recycled water from the Howard F. Cullen WWTP through the Tampa Bypass Canal was selected as the optimum system.	[7,28]
San Diego Water Repurification Project	California (USA)	1981	In 1985 Several treatments tested including RO and granular activated carbon. Since 2002 MF/RO, and advanced oxidation using UV light and hydrogen peroxide.	Reservoir	NA	NA	Demonstration project between 1985–1999 and since 2002 full-scale project for non-potable reuse only due to community opposition. Health effects study conducted in 1985.	[27]
Potomac Estuary Experiment al Wastewater Treatment Plant (EEWTP)	Washington D.C. (USA)	1980–1982	Floculation, sedimentation, filtration, granular activated carbon adsorption and disinfection.	Estuary	NA	NA	Two years demonstration project. The EEWTP influent water was 50% recycled water and 50% estuary water. The EEWTP blended water treated with conventional drinking water process (such as: flocculation, sedimentation and disinfection) followed by granular activated carbon and chlorination.	[7,30]
Hueco Bolson Recharge Project	Texas (USA)	1985	Two-stage powdered activated carbon treatment, lime treatment, two-stage recarbonation, sand filtration, ozonation, GAC filtration, chlorination, and storage.	Aquifer	250,000	40–100%	Full-scale project.	[92]
The Chelmer Augmentati on Wastewater Reuse Scheme (Water 2000)	Essex England	1997	MF UV	Reservoir	1.7 million	8–12%	Recycled water discharged into the Chelmer river which is used to augment the Hanningfield reservoir. The reservoir storage time is up to 214 days Monitoring of viruses and estrogens since 1996. Hormones in reservoir <LOD of 3 ng/L	[81] [93]
Water Reclamatio n Study (NeWater)	Singapore	2000	Ultrafiltration, RO, UV, Stability control and chlorination	Reservoir	4.4 million	Currently 1% and 2.5% by 2012	Initially a demonstration plant, but has operated as a full-scale plant since 2002 when adoption for augmentation of drinking water supplies was recommended. Full-scale project with 3 existing plants. Total production of 92 ML/day from 3 plants. The majority of recycled water is used for industry. Project supported by a well designed community education program.	[12,23]
Goreangab Water Reclamatio n Plant	Windhoek Namibia	1968–2002 Upgrade 2002– present	Algae flotation Foam fractionation Chemical clarification Sand filtration Granular activated carbon Chlorination Pre-ozonation for Fe/Mn removal Dissolved air flotation Sand filtration Ozonation Granular activated carbon Ultrafiltration Chlorination	Reservoir		4% 25%	Sometimes used for direct potable reuse.	[94,95]
Torreele Reuse Plant	Wulpen Belgium	2002	MF/RO + UV disinfection	Aquifer	60,000	40%	Full-scale project that produces between 40 to 50% of the drinking water demand. The minimum retention time in the aquifer is 40 days. Reported improvement in drinking water quality with lower hardness and better color due to decreased organic content.	[11,96]

Year: year project started; % blended: % of recycled water blended with alternate sources; Population: population served in the distribution area

**Table 2.** Epidemiological studies direct and indirect potable reuse projects.

Project	Aim of the study	Study years	Experimental Details	Results	Source
Montebello Forebay Groundwater Recharge Project Health Effects Study No 1	Assessment of health outcomes between the Montebello Forebay area, which has received some recycled water in its water supply with a control area.	1969–1980	Descriptive, ecological study of more than a million people. Four recycled water exposure categories (high, low and two control groups), although the variable proportion of recycled water in the study area led to issues of exposure misclassification. Three time periods compared: 1969–1971, 1972–1978 and 1979–1980. The study did not account for several confounding factor The Scientific Advisory Panel in 1986 concluded that cancer outcomes were inconclusive due to high mobility of the population and long latent period for human cancers. The short and long term effects studied included mortality, infectious diseases, adverse birth outcomes and cancer incidence. An additional household survey in 1981 interviewed 2523 women for information on reproductive outcomes and water consumption.	The population ingesting recycled water did not demonstrate any measurable adverse health effects. However, the Scientific Advisory Panel in 1986 concluded that cancer outcomes are inconclusive due to high mobility of the population and long latent period for human cancers. The household survey found no differences on specific illnesses or measures of general health between participants living in high and low recycled water areas. No association were found for low birth weight, infant mortality or congenital malformations.	[7,24]
Montebello Forebay Groundwater Recharge Project Health Effects Study No 2	Assessment of health outcomes between the Montebello Forebay areas, which has received some recycled water in its water supply for almost 30 years, with a control area.	1987–1991	Ecological study of a population exposed to between 0 and 31% recycled water over a 30-years (1960–1991). Five exposure categories (four groups receiving increased percentages of recycled water and one control group) although variable proportion of recycled water in the study area with issues of exposure misclassification. Multivariate Poisson regression used to generate rate ratios. The study did not account for many confounding factors	No evidence that recycled water has an adverse effect on cancer incidence, mortality and infectious disease outcomes. Significantly higher incidence rate of liver cancer in the area with the highest percentage of recycled water was observed. However, due to limitations of the study and the lack of dose-response trend the authors conclude that the results are more likely explained by chance or unaccounted confounding variables.	[19]
Montebello Forebay Groundwater Recharge Project Reproductive Study	Assessment of adverse health outcomes among live born infants, including low birth weight, preterm births, infant mortality and 19 categories of birth defects.	1982–1993	A cohort study that extended the original reproductive outcomes conducted in 1981. Exposure group allocation based on the average annual percentage of recycled water in water supplied by the systems serving the ZIP- code. Place of residence was used as surrogate measure for exposure which may over-estimate or sub-estimate the true exposure scenario and no data on individual exposure was collected. High population mobility may decrease the validity of the results. The study did not account for several confounding factors such as smoking or alcohol consumption but is assumed to be equal between the recycled water and control groups.	The study does not provide evidence of an association between recycled water and adverse birth outcomes. Rates of adverse outcomes were similar in groups receiving high or low percentages of recycled water.	[20]
Potable Reuse Project Windhoek (Namibia)	Assessment of cases of diarrhoeal diseases, jaundice, and deaths in Windhoek, where the average contribution of recycled water to the waster was 4% between 1968 and 1991.	1976–1983	An ecological study of 3000 deaths, excluding pre-natal and unnatural causes of death. Deaths were classified by cause and race. Windhoek statistics were compared to global statistics because Namibian data was not available.	No association between any of the studied health outcomes and drinking water source was found. * Diarrhoea was associated with socio-economic status but not with the recycled water.	[97,98]

**Table 3.** Toxicological studies indirect potable reuse projects.

Project	Aim of the study	Experimental Details	Results	Source
Orange County Water District. Water Factory 21 Santa Ana River Water Quality and Health Study (Evaluation Task No 7)	Water quality evaluation and risk assessment of Santa Ana River, imported water and recycled water from Water Factory 21. At the time of the study more than 90% of the base flow of the Santa Ana River comprises wastewater discharge which is the primary source for recharging the groundwater basin	The relative risks to human health associated with the three water sources (Santa Ana River, imported water or recycled water) were compared using the USEPA drinking water guidelines. Quantitative relative risk assessment methods used to compare the water sources. Estimates of the relative risk to human health associated with each water source were calculated. For the microbial assessment it was assumed that each water source was consumed directly before being used to recharge the groundwater basin. Risk assessment was reviewed by an independent Scientific Advisory Panel to assess the Santa Ana River Water Quality and Health Study in 1996. The Committee agreed with the report’s conclusions and concluded that the health risk associated with the quality of the recycled water will be equal or less than the other two water sources	Most of the organic carbon in the river and recharge basins is of natural origin and no chemicals of wastewater origin were identified at concentrations of public health concern. Anthropogenic dissolved organic carbon (20–25% of total DOC) consisted mostly of detergents and surfactants. None of the three water sources posed significant non-carcinogenic risk to public health and the risk posed by recycled water was lower than the other sources. Similarly the carcinogenic risk associated with direct consumption of recycled water was lower than the associated with the other sources. NDMA and 1,4-Dioxane are the constituents that present more carcinogenic risk in recycled water, while NDMA at an assumed maximum concentration of 20 ng/L presented the highest carcinogenic risk. Water produced by MF/RO treatment was safe for consumption and actually improved the groundwater basin’s water quality. Recycled water at the point of recharge is projected to pose much less of a risk for bacteria, parasites and virus than the other water sources as long as all unit processes in the treatment are operating properly. Arsenic is the analyte that accounts for the majority of risk in all water sources.	[29,99]
Denver Potable Water Demonstration Project	Chronic toxicity and oncogenicity studies in animals.	Toxicological studies evaluated: clinical observations, survival rate, growth, food and water consumption, haematology, clinical chemistry, urinalysis, organ weights, gross autopsy and histopathology of major tissues and organs. Fischer 344 rats and B6C3F1 mice were exposed to 150-fold and 500- fold recycled water concentrates for up to 2 years. Sprague-Dawley rats were used for reproductive studies.	Clinical pathology, gross pathology, and microscopic pathology conducted at weeks 26 and 65 and at the end of the study did not reveal any differences that could be considered to be treatment related. No adverse health effects were detected from lifetime exposure to any of the samples and during a two-generation reproductive sample.	[100, 101]
Orange County Water District GWR system	On-line biomonitoring of fish to evaluate the water quality.	Shallow ground water originating from the Santa Ana River (approximately 85% of the river base flow comes from recycled water) and constituted control water compared in a 9 months experiment. Japanese medaka used as bioindicator Recycled water and treated recycled water with granular activated carbon were also compared in a 3 months experiment.	No statistically significant differences in gross morphological endpoints, overall mortality, gender ratios histopathology or reproduction were observed in the 9 month study. * In the 3 months experiment reproduction and exposure to bio- available estrogenic compounds was evaluated with no significant differences observed between treatments.	[78]
Denver Potable Water Demonstration Project	Chronic toxicity and oncogenicity studies in animals.	Clinical observations, survival rate, growth, food and water consumption, haematology, clinical chemistry, urinalysis, organ weights, gross autopsy and histopathology of major tissues and organs were evaluated. Fischer 344 rats and B6C3F1 mice were exposed to 150-fold and 500- fold recycled water concentrates for up to 2 years. Sprague-Dawley rats were used for reproductive/teratology studies.	Clinical pathology, gross pathology, and microscopic pathology conducted at weeks 26 and 65 and at the end of the study did not reveal any differences that could be considered to be treatment related. No adverse health effects were detected from lifetime exposure to any of the samples and during a two-generation reproductive sample.	[100,101]
Denver Potable Water Demonstration Project	Water quality assessment Organic challenge study.	Recycled water was compared with the drinking water. Fifteen organic compounds were dosed at approximately 100 times the normal levels found in the reuse plant influent.	The recycled water quality was better than the Denver drinking water for all chemical, physical, and microbial parameters tested except for nitrogen, and alternative treatment options were subsequently implemented for nitrogen removal Challenge study demonstrates that the multiple-barrier process can remove most of tested contaminants to non-detectable levels. RO effluent met drinking water standards for all pathogens sampled, but failed to meet drinking water standards for a few contaminants.	[28]
Hueco Bolson Recharge Project	Water quality assessment	Routine sampling program implemented.	Bacteriological tests have shown an average total of zero coliform per 100 mL of effluent water. The existing priority pollutant monitoring of the injection well system has detected only trihalomethanes, at levels below the USEPA limit of 100 *μg*/L	[28]
Montebello Forebay Groundwater Recharge Project (Health Effects Study)	Characterization of water quality for microbiological and inorganic chemical content. Toxicological and chemical studies to isolate and identify organic constituents of significance to health.	Five year study starting in 1978 called Health Effects Study compared the quality of groundwater, recycled water, storm water and imported water. Ames *Salmonella* test and mammalian cell transformation assay were performed on all waters as well as recycled water concentrate 10,000 to 20,000 times, with subsequent chemical identification. At the time of the study approximately 16% of the injected water was recycled water.	Concentrations of industrial organics and metabolic by-products such as phthalates, solvents and petroleum by- products were higher in recycled and storm waters but below EPA standards. No relation was observed between % of recycled water in wells and observed mutagenicity of residues isolated from wells. The proportion of recycled water currently used for replenishment had no measurable impact on either groundwater quality or human health. None of 174 samples tested positive for viruses. Only 10% of the organic matter contained in the recycled water could be characterised. Mutagenic activity using Ames test and *Salmonella* tester strains (TA98 and TA 100) was detected in 43 of 56 samples tested, including at least one from each source, and was attributed to chlorinated compounds. The level of mutagenic activity (in decreasing order) was storm runoff > dry weather runoff > recycled water > ground water > imported water.	[20,24,30]
Water Reclamation Study (NeWater) Health Effects Study	Water quality and toxicological studies.	NeWater was compared to raw and drinking water in the water quality- monitoring program in which more than 190 physical, chemical and microbiological parameters were tested. • The mice strain (B6C3F1) was used for chronic toxicity and carcinogenicity. Mice were fed for up to 2 years with 150x and 500x concentrates of NeWater and reservoir water. * A year-long fish study conducted to assess long-term chronic toxicity and estrogenic effects using the orange-red Japanese medaka fish.	All tested parameters were below WHO and USEPA drinking water guidelines and standards for both NeWater and drinking water. The 3 and 12 month results indicated that exposure to concentrated recycled water did not cause any tissue abnormalities or health effects. The 24 months results remain unpublished. No estrogenic or carcinogenic effects reported in the fish studies.	[23]
San Diego Water Repurification Project	Water quality assessment	Twenty-nine endocrine disrupter, pharmaceuticals and personal care products tested. Triclosan detection after advanced oxidation was possible due to bottle contamination.	Low-level concentrations of trihalomethanes were detected below drinking water standards. Eight of 29 emerging contaminants were detected after RO but only triclosan remain after advanced oxidation.	[28,30]
Tampa Water Resource Recovery Project (Health Effects Study)	Characterization of water quality for chemical, physical and microbiological content. Toxicological testing	Recycled water quality was compared to raw water from the Hillsborough River. Raw water was disinfected with ozone before analysis to make it more analogous to the recycled water. Toxicological testing of recycled water produced from 4 different processes was compared in 1992. Toxicological testing used up to 1000x organic concentrates used in Ames *Salmonella,* micronucleus, and sister chromatid exchange tests in three dose levels. In addition a 90 day sub chronic assay and developmental studies were performed on mice and rats, and reproductive toxicity was studied in mice only. *In vivo* testing included mouse skin initiation (SENCAR mice initiation- promotion studies) and strain A mouse lung adenoma.	The recycled water did not present significant microbiological or toxicological risks. Viruses were detected in 6.7 % of the samples after chlorination, but this occurred during an operational period when pH levels were suboptimal. Mutagenic activity tested using *Salmonella/microsome* assay was positive but no significant positive response was observed *in vivo.* All tests were negative for developmental toxicity, except for some foetal toxicity exhibited in rats, but not mice, for the advanced water treatment sample A panel of six internationally recognized water quality and health effects experts comprised a Health Effects Group that concluded recycled water is safe for human consumtion.	[7,28]
San Diego Water Repurification Project. (Health Effects Study)	Identification, characterization and quantification of infectious diseases agents and potentially toxic chemicals. Screening for mutagenicity and bio- accumulation of chemical mixtures. Chemical risk assessment.	Study compared the genetic effects of recycled water and the existing raw water supply. 150–600x organic concentrates were used in Ames *Salmonella* test; micronucleus, 6-thioguanine resistance, and mammalian cell transformation testing were conducted. Biomonitoring experiments using fathead minnows and fish to evaluate survival, growth, swimming performance and chemical bio-accumulation conducted. Trace amounts of 68 base/neutral/acid extractable organics, 27 pesticides, and 27 inorganic chemicals were tested in fish tissues after exposure.	The average total organic carbon concentration was 1.37 mg/L in the recycled water and 9.83 mg/L in the raw water. Similar inorganic species were found in samples from both waters, although there was greater evidence of bio-accumulation from raw water. The Ames test showed some mutagenic activity, but recycled water was less active than drinking water. The micronucleus test showed positive results for both waters but only at the high (600x) doses than for raw water. *In vivo* fish biomonitoring (28-day bio- accumulation and swimming tests) showing no positive effects. Recycled water and raw water were only distinguishable in 28 days chemical bio- accumulation tests for pesticide levels, which were higher in raw water. Better performance of fish survival, growth, and swimming performance after 90 and 180 days exposure in the raw drinking water may be related to ionic composition. There was no significant health risk from non-carcinogenic chemicals in either water. The chemical risk estimates were dominated by bis(ethylhexyl)phthalate in recycled water and by arsenic and trihalomethanes in the raw water. The risk from human intake of recycled water was 40 times lower	[27,30,102,103]
Potomac Estuary Experimental Wastewater Treatment Plant	Toxicological studies	Water quality achieved from the blending of 50% recycled water after secondary treatment and 50% Potomac estuary water was compared with drinking water. Ames *Salmonella* test and mammalian cell transformation assay were conducted using organic concentrates of 150-fold. * The NRC report did not support the study conclusion due to few toxicological studies conducted.	Recycled EEWTP water had less mutagenic activity (the effluent tested positive only about 10 percent of the time) than the drinking water by the Ames test. The cell transformation assays also tested positive for both waters with similar small numbers of positive results. The study concludes that the treatment produce a water quality acceptable for human consumption, although the National Research Council report did not support the study conclusion due to the limited number of toxicological studies conducted.	[7,30]
